# Serial and parallel convolutional neural network schemes for NFDM signals

**DOI:** 10.1038/s41598-022-12141-4

**Published:** 2022-05-13

**Authors:** Wen Qi Zhang, Terence H. Chan, Shahraam Afshar Vahid

**Affiliations:** 1 Laser Physics and Photonic Devices Laboratories, STEM, University of South Australia, Adelaide, Australia; 2 Institute for Telecommunications Research, University of South Australia, Adelaide, Australia

**Keywords:** Nonlinear optics, Fibre optics and optical communications

## Abstract

Two conceptual convolutional neural network (CNN) schemes are proposed, developed and analysed for directly decoding nonlinear frequency division multiplexing (NFDM) signals with hardware implementation taken into consideration. A serial network scheme with a small network size is designed for small user applications, and a parallel network scheme with high speed is designed for places such as data centres. The work aimed at showing the potential of using CNN for practical NFDM-based fibre optic communication. In the numerical demonstrations, the serial network only occupies 0.5 MB of memory space while the parallel network occupies 128 MB of memory but allows parallel computing. Both network schemes were trained with simulated data and reached more than 99.9% accuracy.

## Introduction

Nonlinear Fourier Transform (NFT) has been proposed as an alternative technique for fibre optics communication to break through the Shannon linear capacity limit due to the nonlinearity of optical fibres^1–4^. It has already been experimentally demonstrated that NFT can outperform conventional linear Fourier transform-based systems^5–7^. However, NFT-based communication methods have not yet reached the maturity level of conventional methods. One reason for this is the complexity of NFT calculations, where speed and accuracy are the two main bottlenecks for practical applications^8,9^. Despite the development of fast NFT algorithms^10–13^ which has improved the speed of NFT calculation, the lack of hardware implementations of NFT due to algorithm complexity remains a challenge. Very recently, a conceptual hardware implementation has been proposed on an FPGA platform^14^. However, due to the complexity of NFT algorithms, implementing and optimizing hardware designs require a lot of effort before any products can be built for practical use and with perform better than existing conventional devices.

In recent years, machine learning (ML) has demonstrated great success in many areas of science and technologies. Although the development of ML for optical communication is still in its early stages, ML has shown potentials for solving many challenges^15–21^, including the field of NFT-based communication^22–29^. At the same time, due to the popularity of machine learning, hardware implementation of deep neural networks has become an active research area with supports from mega-corporations such as Google and Nvidia^30–36^. Hence, there is an opportunity to take advantage of the rapid development of deep learning hardware and use it for NFT applications.

Early work of using ML for NFT-based communication was mainly focused on post-processing, such as constellation classification^25^ and equalisation^23,26^, instead of replacing the direct NFT calculation. An attempt of using a neural network to decode nonlinear QPSK modulation was carried out by Jones et al.^22^, but the development of the network was limited only to second-order soliton pulses. Recently, a convolutional neural network (CNN) was proposed to directly decode nonlinear frequency division multiplexing (NFDM) signals with complexity (16-QAM with 128 subcarriers) which was comparable to experimental setups^29^. However, in this previous work, the network was not used efficiently, especially for signals with many subcarriers, since for every subcarrier, a separate network was needed. Furthermore, the previous work only demonstrated networks for a fixed signal power without considering fibre loss and in the case of lump amplification. While this paper was being reviewed, two new articles were published^27,28^. Both of them were aimed at obtaining the nonlinear spectrum of a signal. In the first article^27^, two CNN sub-structures were applied to process the real and imaginary parts of the signal in parallel. However, the training data used in this work only has 15 subcarriers and four possible choices for each subcarrier. Therefore, the scalability of this proposed network is also unknown. Furthermore, the training data does not include variation in signal propagation distance, absorption loss and variation in power, hence the generality is hard to evaluate. In the second work^28^, a network architecture similar to the one in the work^29^ is used for both direct and inverse NFT. However, this article did not give any details on the training data. Hence, the generality and scalability of their network cannot be evaluated.

In this paper, we develop two network design ideas with hardware implementation kept in mind; a serial scheme aimed at a small network size and a parallel scheme aimed at high speed. In each scheme, a custom transformation layer is designed using the properties of NFT that enables the network to work with signals at different propagation lengths and power levels. An optical pulse propagation model is used to simulate signal propagation in optical fibre including the effects of fibre nonlinearity, chromatic dispersion, absorption loss, Raman scattering and lump amplification to closely mimic realistic experimental settings. Using the simulation, we generated 128-subcarrier 16-QAM signals with variable propagation length and power level for network training and validation.

The paper is divided into six sections apart from the Introduction. In the second section, a quick introduction to nonlinear Fourier transform and nonlinear frequency division multiplexing is given. In the next section, the basic parameters and descriptions are given on the generation of the training and validation data. The serial and parallel network schemes are discussed in the following sections. Finally, a conclusion and a method section are given at the end.

## Modulation of continuous nonlinear spectrum

Transforming a temporal signal *q*(*t*) into the nonlinear Fourier domain is achieved by solving the following differential equation^1^

1 $$\begin{aligned} v_{t}=\left( \begin{array}{cc} - i\lambda &{} q(t) \\ -q^{*}(t) &{} i\lambda \end{array} \right) v\text {,} \end{aligned}$$

with the initial condition:

2 $$\begin{aligned} v(t,\lambda )&= \left( \begin{array}{l} v_1(t,\lambda )\\ v_2(t,\lambda ) \end{array} \right) \rightarrow \left( \begin{array}{l} 1 \\ 0 \end{array} \right) e^{-j\lambda t},&\quad t\rightarrow -\infty \end{aligned}$$

where $v_t$ is the temporal derivative of *v* and $\lambda$ is the nonlinear frequency. Two time invariant coefficients $a(\lambda )$ and $b(\lambda )$ can be found using the following limits^3^:

3 $$\begin{aligned} \begin{array}{ccc} a(\lambda )=\displaystyle \lim _{t\rightarrow \infty } v_1(t,\lambda )e^{j\lambda t},&\,&b(\lambda )=\displaystyle \lim _{t\rightarrow \infty } v_2(t,\lambda )e^{-j\lambda t}. \end{array} \end{aligned}$$

The nonlinear spectrum of signal *q*(*t*) is thus defined as

4 $$\begin{aligned} Q(\lambda )&= \left\{ \begin{array}{l} b(\lambda )/a(\lambda ) \quad \text {for} \quad \lambda \in {\mathbb {R}}\text {,}\\ b(\lambda )/a^\prime (\lambda ) \quad \text {for} \quad \lambda \in \mathbb {C}^+ \text { and } a(\lambda )=0\text {,} \end{array} \right. \end{aligned}$$

where $\displaystyle a^\prime (\lambda )=\frac{\partial a(\lambda )}{\partial \lambda }$. The nonlinear Fourier spectrum is continuous for $\lambda \in {\mathbb {R}}$ and discrete when $a(\lambda )=0$ for $\lambda \in \mathbb {C}^+$. The spectrum in both continuous and discrete region can be used to carry information, but in this work, we focus on the continuous part only.

Nonlinear frequency division multiplexing (NFDM) is a multiplexing technique borrowed from orthogonal frequency division multiplexing (OFDM) in convectional fibre optic communication, in which the linear spectrum of OFDM is replaced by a nonlinear spectrum^3^ and becoming popular in recent years^6,37–41^. The modulation is usually applied to the continuous part of the nonlinear spectrum $Q(\lambda )$ directly, but modulating $b(\lambda )$ (b-modulation) has also been used as it results in pulses with well defined temporal windows^7,42–46^.

The modulation can often be expressed as

5 $$\begin{aligned} s(\lambda )=\sum _n c_n w_n(\lambda )\text {,} \end{aligned}$$

where *n* is the number of subcarriers, $c_n$ is the complex data symbols, $w_n(\lambda )$ is the carrier wave. Conventionally, $\text {sinc}$ functions are used as $w_n(\lambda )$^37^. Other choices of functions such as raised cosine or a flat-top window with cardinal sine carrier waveform have also been proposed^43^. The function $s(\lambda )$ is the modulated spectrum. It is often scaled to match the desired signal power. For Q-modulation case, $Q(\lambda )=s(\lambda )$. An inverse NFT can be performed to obtain the temporal signal *q*(*t*)^37^. In the case of b-modulation, $b(\lambda )=s(\lambda )$, and an additional step is needed to generate $a(\lambda )$ from $b(\lambda )$ before the inverse NFT step can be taken^42^.

Inspired by the work^43^, we used the following carrier wave functions for this work.

6 $$\begin{aligned} w_n(t)= &\, \text {sinc}\left( 4\frac{t}{T_0}\right) \cdot \text {exp}\left[ -2\left( \frac{t}{\tau T_0}\right) ^2-i n\pi \cdot 8\frac{t}{T_0}\beta \right] \end{aligned}$$

7 $$\begin{aligned}= &\, \text {sinc}\left( 4\frac{t}{T_0}\right) \cdot \text {exp}\left[ -2\frac{t}{T_0}\left( \frac{t}{\tau ^2 T_0}+i 4n\pi \beta \right) \right] \text {,} \end{aligned}$$

where $T_0$ denotes the width to the 4th zero of the $\text {sinc}$ function [note: here $\text {sinc}(t)=\sin (\pi t)/(\pi t)$], $\tau$ is a free parameter that adjusts the width of the Gaussian, which also related to the sharpness of the flat-top waveform in the frequency domain, and $\beta$ adjusts the separation between subcarriers.

In the nonlinear frequency domain, the corresponding $w_n(\lambda )$ can be obtained by replacing the angular frequency in $w_n(\omega )$ with $2\lambda$ (or $-2\lambda$ depending on the choice of the sign in the Fourier transform), where $w_n(\omega )$ is the linear Fourier transform of $w_n(t)$;

8 $$\begin{aligned} w_n(\lambda )=\frac{T_0}{8\sqrt{2\pi }}\left\{ \text {erf}\left[ \frac{\tau T_0}{\sqrt{2}}\left( \lambda -\frac{4\pi \beta }{T_0}n+\frac{2\pi }{T_0}\right) \right] -\text {erf}\left[ \frac{\tau T_0}{\sqrt{2}}\left( \lambda -\frac{4\pi \beta }{T_0}n-\frac{2\pi }{T_0}\right) \right] \right\} \text {.} \end{aligned}$$

From Eq. (8), one can see the nonlinear frequency separation between subcarriers is $4\pi \beta /T_0$, whilst $4\pi /T_0$ is approximately the bandwidth of each subcarrier. For the rest of the work, $\tau =1$ and $\beta =1$ are used.

## Generation of training data

The preparation of the training data is crucial to the performance of a neural network. Ideally, the training data should be generated experimentally such that the network can be tuned to the specific experimental setup. However, for demonstration purposes, we generate the training data through numerical simulation while the simulation conditions are tuned to match those of the experiments closely. Assuming a signal generator with a sampling rate of 96 GS/s with total electronic 3-dB bandwidth of 28 GHz. A time window of 21.33 ns is used for each burst of the signal. The laser source has a line-width of 100 Hz at the central wavelength of 1550 nm. Standard single-mode fibres are used with the propagation loss of 0.2 dB/km, group velocity dispersion of − 21.7 ps$^2$/km ($\beta _3$ = 18.6 ps$^3$/km) and nonlinearity of 1.1 W$^{-1}$ km$^{-1}$. A maximum of 10 optical fibre spans with 50 km per span are used in the simulation with point amplification at the end of each span. For each burst of signal, a random number of spans between 1 and 10 is chosen. Lump amplification is applied in the simulation and a path-average model is used to counteract the effects of fibre loss and lump amplification^47^. A quadrature amplitude modulation is used (16-QAM) for encoding subcarriers with direct mapping of numbers 0–15 to the symbols. The numbers are then used as labels for the network training algorithm.

We desire to have the network perform equally at different input power levels in the range of −20 to 0 dBm, hence we need a way to randomly generate the training data with power evenly distributed within this range to avoid bias of the final network towards certain input power. To achieve this, we have developed and used an approximated energy calibration model, which is discussed in the next section. The noise background in the training data is chosen to be −40 dBm per amplification.

### An approximated model for energy calibration

The energy of a burst can be evaluated using its nonlinear spectrum $Q(\lambda )$ as

9 $$\begin{aligned} E=\frac{1}{\pi }\int {\ln (1+\left| A\cdot Q(\lambda )\right| ^2)d\lambda }\text {,} \end{aligned}$$

where *A* is a real scaling factor through which different burst energy can be set. The energy *E* does not scale linearly with *A*. Depending on the choice of the carrier wave and the number of subcarriers, the same *A* results in different energy levels. This is particularly difficult for generating training data since a uniform distribution of power on the log scale is needed (−20 to 0 dBm in this work). Here we present a fitting model that gives an approximated mapping between *A* and the average burst energy.

Firstly, we discretize the integral in Eq. (9) as

10 $$\begin{aligned} E=\frac{1}{\pi }\sum _i\ln \left( 1+A^2\left| Q_i\right| ^2\right) \Delta \lambda \end{aligned}$$

where *i* changes from 1 to the number of discrete points across the nonlinear spectrum. Rearranged the equation, we have

11 $$\begin{aligned} E\frac{\pi }{\Delta \lambda }= & {} \sum _i\ln \left( 1+A^2\left| Q_i\right| ^2\right) \end{aligned}$$

12 $$\begin{aligned}= & {} \ln \left[ \prod _i\left( 1+A^2\left| Q_i\right| ^2\right) \right] \text {.} \end{aligned}$$

Let $C=\prod _i\left( 1+A^2\left| Q_i\right| ^2\right)$ and take natural logarithm on both sides of the equation above, we have

13 $$\begin{aligned} \ln {E}+\ln \frac{\pi }{\Delta \lambda }=\ln {\ln {C}}\text {.} \end{aligned}$$

Next, we normalize the symbol constellation to its mean amplitude, $c_n/\bar{\left| c_n\right| }$, and the carrier wave function to its peak amplitude, $w_n(\lambda )/\text {max}\left( w_n(\lambda )\right)$. From now on, all $Q(\lambda )$ shall be generated using the normalized constellation and carrier waves. After that, we assume a new constant $|{\bar{Q}}|$ such that

14 $$\begin{aligned} E\frac{\pi }{\Delta \lambda }= & {} \ln \left[ \prod _i\left( 1+A^2\left| Q_i\right| ^2\right) \right] \end{aligned}$$

15 $$\begin{aligned}= & {} \ln \left( 1+A^2\left| {\bar{Q}}\right| ^2\right) ^n \end{aligned}$$

16 $$\begin{aligned} E\frac{\pi }{\Lambda }= & {} \ln \left( 1+A^2\left| {\bar{Q}}\right| ^2\right) \text {,} \end{aligned}$$

where *n* is the number of samples in $\lambda$ and $\Lambda =n\Delta \lambda$ is the window size in the nonlinear spectrum. Now, we define a new parameter $A^\prime$ such that

17 $$\begin{aligned} A^\prime \left| 1\right| =A\left| {\bar{Q}}\right| =\sqrt{e^{\left( \displaystyle \frac{\pi }{\Lambda }E\right) }-1}\text {.} \end{aligned}$$

**Figure 1:**
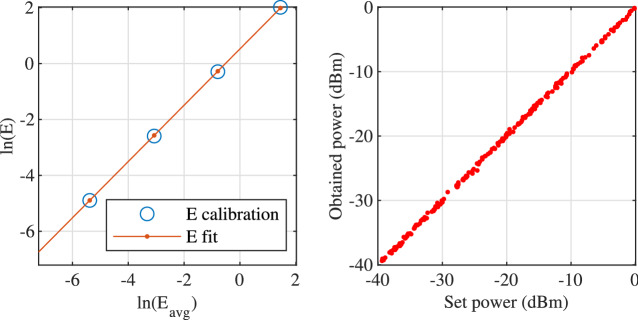
Power calibration model. Left: linear fitting of the model. Right: 200 randomly generated pulses using the energy calibration model.

And we choose a few points $E^\prime$ within the energy range $[E_\text {min},E_\text {max}]$ to calculate $A^\prime$ using Eq. (17). Once, we get $A^\prime$, we replace *A* in Eq. (9) with $A^\prime$ and calculate the actual averaged pulse energy $E_\text {avg}$ using randomly generated $Q(\lambda )$. Depending on the choice of carrier wave function, QAM format, number of subcarriers, etc., $E_\text {avg}$ differs from $E^\prime$ Figure 1 shows an example.

Finally, a least square fit is applied to $\ln E_\text {avg}$ and $\ln E^\prime$,

18 $$\begin{aligned} \left( \begin{array}{c} p_1\\ p_0\end{array}\right) =\text {pinv}\left( \left[ \begin{array}{lr} \ln E_\text {avg}&J \end{array} \right] \right) \times \ln E^\prime \text {,} \end{aligned}$$

where $\ln E_\text {avg}$ and $\ln E^\prime$ are column vectors, *J* is an all-ones column vector and $\text {pinv}$ is the matrix pseudo-inverse function. And finally the calibrated $A_\text {cal}$ is calculated as

$$\begin{aligned} A_\text {cal}=\sqrt{e^{\displaystyle \frac{\pi }{\Lambda }\left( p_1 \ln E + p_0\right) }-1}\text {.} \end{aligned}$$

## The serial CNN scheme

In the original work^29^, to decode each subcarrier, a transformation is needed to be performed on the received signal to remove the propagation induced temporal shift and phase change. Then, each subcarrier is decoded using its corresponding network. This process is inefficient since the differences between the nonlinear spectra of subcarriers are rather small, except for the ones on the edges of the nonlinear spectrum. There are overlaps between the networks from previous work and in principle, all of them can be combined into one. Furthermore, during our study, we realised that the evolution of the linear spectrum of subcarrier *k* is mostly affected by its neighbouring subcarriers. Therefore, the input to the network can be reduced significantly by applying a gate function to the input pulse in the linear frequency domain, hence greatly reduce the parameter space of the network.

**Figure 2:**
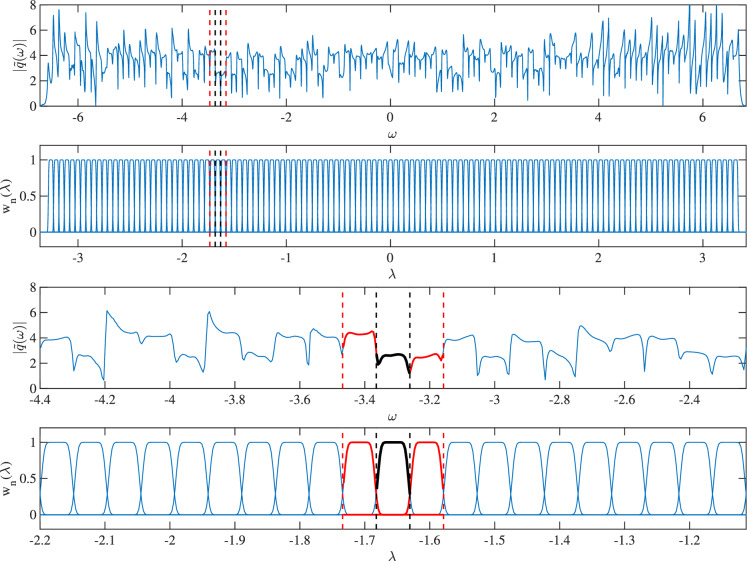
An example signal is shown in the linear frequency domain compared with subcarriers waves in the nonlinear frequency domain (top two figures). Two red vertical lines indicate the gated pulse that is used for decoding. The two black vertical lines indicate the subcarrier to be decoded by the network. Bottom two figures are the zoomed in view of the top figures, respectively, for clarity.

Figure 2 illustrates the idea. An example pulse in the linear frequency domain is aligned with 128 subcarrier waves $w_n(\lambda )$, where the nonlinear frequency is half of the linear frequency ($2\lambda =\omega$). For any arbitrary subcarrier (between two black vertical lines), one adjacent subcarrier on each side (between two red lines) is used as the input to the network.

With these ideas in mind, we design a serial network scheme that works with all subcarriers. A network is trained for only 1 subcarrier (subcarrier $n=0$). To decode any other subcarriers using this network, a segment of the pulse spectrum $q(\omega )$ around a subcarrier (between the two red lines) is taken out and shifted using Eq. (19);

19 $$\begin{aligned} \tilde{q}_k(\omega ,z)=\tilde{q}(\omega -2\lambda _k,z) e^{j t_k (\omega -\lambda _k)} \text {,} \end{aligned}$$

where $\lambda _k$ is the central nonlinear frequency of subcarrier *k*, $t_k=4 \lambda _k z$. This equation shifts the central frequency of subcarrier *k* to subcarrier 0 and removes the phase change due to propagation from the nonlinear spectrum^29^. Note that a negative sign is used in the Fourier transforms when getting $\tilde{q}(\omega )$ from *q*(*t*). The shift of Eq. (19) is built into the network as a transform layer.

**Figure 3:**
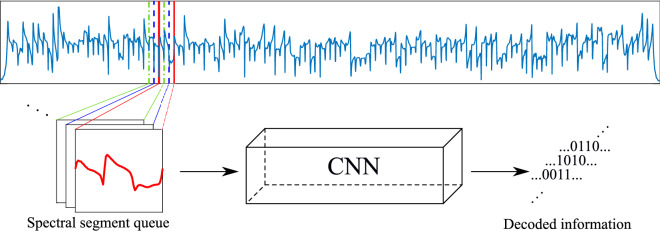
Illustration of the serial network decoding scheme. Segments of the spectrum corresponding to every subcarrier are queued up and passed through the network in serial. The spectra between the red solid, blue dashed and green dash-dotted lines are the segments of adjacent subcarriers in a queue.

All the subcarrier segments are collected in a queue and feed into the network. Figure 3 illustrates the idea. For the first and last subcarrier, zero paddings are applied. The output of the network is also a queue in the same order as the input. For each subcarrier input, the network outputs the decoded data corresponding to the mapping of the symbol $c_n$. In this work, it is number 0–15.

**Figure 4:**
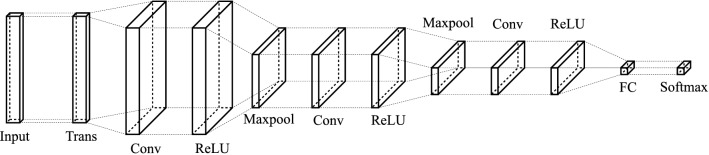
The conceptual schematic of the serial network. *Trans* transform layer, *Conv* convolutional layer, *ReLU* rectified linear unit activation layer, *Maxpool* max-pooling layer, *FC* fully connected layer, *Softmax* softmax layer. The number of convolution, activation and pooling layers can be added or removed depending on the input signal.

The schematic of the CNN is shown in Fig. 4. In the figure, only three convolutional layers are shown. However, depending on the input signal, e.g. the carrier wave function and the width of the spectrum segment, the network depth can be adjusted to maximize the network accuracy.

The network used in this work contains 4 convolutional layers. The input to the network is a 32-rows long by 2-columns wide array with each column corresponding to the real and imaginary parts of the 32 sample points of the input segment. Equation (19) is implemented into the network as a transform layer. All the convolutional layers have 64 kernels with a size of $3\times 3$ for the first layers and $3\times 1$ for the rest of the layers.

**Figure 5:**
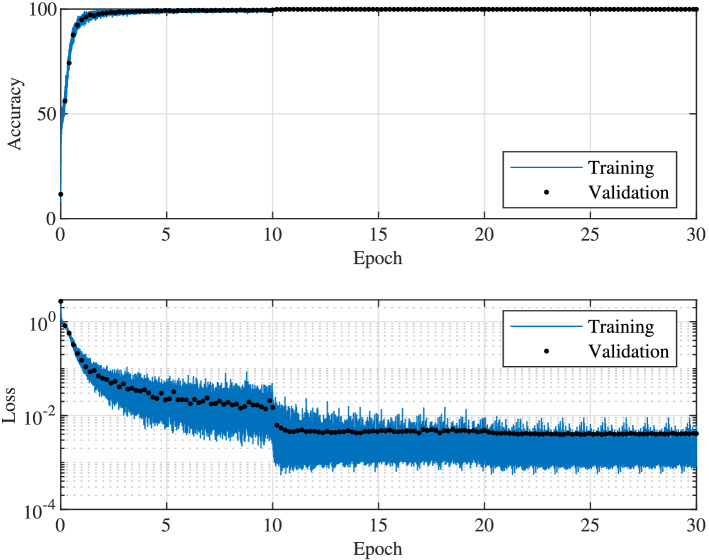
The accuracy and loss as functions of epochs for the serial network scheme. Learning rate changes take place at epoch 11 and 21.

The network is trained using the Adam optimizer^48^ with L2 regularization. 10,000 simulated pulses were generated within which 99% is used for training and 1% is used for validation. Since each pulse is sliced into 128 segments, the size of validation dataset is 12,800 samples. The decay rate of gradient and squared gradient moving averages of the Adam optimizer are set to be 0.9 and 0.99, respectively. The L2 regularization factor is set to 0.00002. Cross-entropy is used in the training algorithm as the loss function^49^. As shown in Fig. 5, the network converges rather quickly. The accuracy of the network, defined as the percentage of correctly predicted symbols in the total number of symbols, reaches 90% in less than a single epoch and reaches over 99% before epoch 5. The learning rate for the first 10 epochs is set to 0.002 and then reduced to 0.0002 for the next 10 epochs as the convergence has slowed down significantly at epoch 10. A clear jump in the loss plot reflects the reduction of learning rate. The accuracy approaches 99.9% after the reduction. The learning rate was further reduced to 0.00002 for the third 10-epoch period but no further accuracy improvement was observed. From the loss value, we notice the step-wise drops at epoch 10 and 20 are corresponding to the change of learning rate. The loss and accuracy saturate after 21 epochs while the training loss is slightly lower than validation loss which indicates the network may have reached its capability limit for further improvements with the available training data. As an indication, we show the signal features that have been learnt by each convolutional layer in the Supplementary Material.

**Figure 6:**
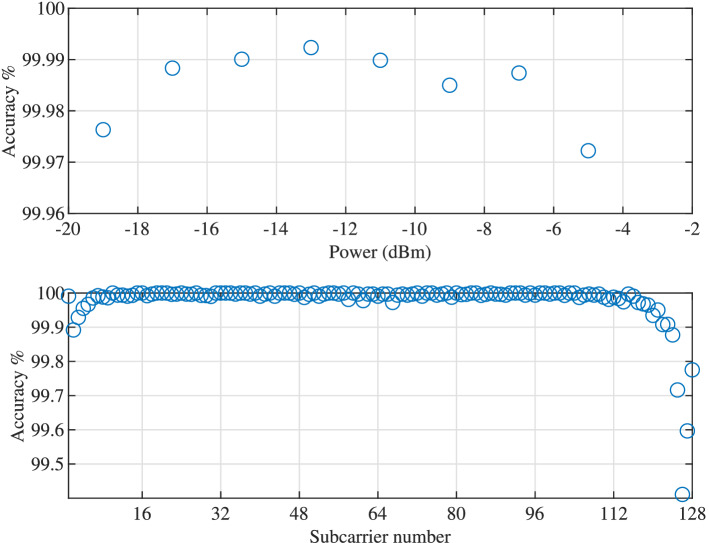
The accuracy for different pulse power and individual subcarrier.

Looking at the network’s prediction accuracy for different pulse power and individual subcarriers as shown in Fig. 6, we notice that the accuracy remains relatively constant for all ranges of pulse power. This can be the result of the fact that the range of power are covered by the training data so that the network is tuned to balance the loss values for different powers. For individual subcarriers, the accuracy is lower on the two edges of the spectrum (subcarrier 2–5 and 117–128), where in the middle, the accuracy is the same and at a high level. Interestingly, subcarrier 1 is on the very edge of the spectrum but still has high accuracy which indicates slight bias in the network toward the first subcarrier. The cause of this phenomenon needs further investigation.

This network scheme optimizes the size of the parameter space. It allows hardware implementation of the network on relatively small chips (the example network only occupies 0.5 MB of memory). Although every subcarrier has to be decoded sequentially, a pipe-lined hardware design is suitable for this network to minimize the speed penalty.

## The parallel CNN scheme

The serial scheme is aimed at small network size, which allows the use of cheap hardware for network implementation with a small speed penalty making it suitable for small end users. But for big end-users, such as data centres, high performance is the major concern instead of hardware costs. Hence, here we purpose a parallel network scheme where all subcarriers can be decoded at once.

In the serial scheme, every subcarrier is taken out of the linear spectrum of the signal with its neighbouring subcarriers before being sent into the network. To decode all subcarriers, the spectral data of each subcarrier is used multiple times in the subsequent decoding process, which can be saved if all the subcarriers can be decoded simultaneously. However, the transformation Eq. (19) is necessary to compensate for the phase change due to propagation. In this section, a new transformation is introduced that allows the design of a multi-output network for simultaneous decoding of all subcarriers.

From Eq. (19) one can see the propagation phase compensation is done through the exponential term. The shift of $\tilde{q}$ in $\omega$ is used to align all the subcarriers. If all the subcarriers are going to be decoded at once, the frequency shift is not necessary. Furthermore, $2\lambda _k$ is the central frequency of subcarrier *k*. To decode all subcarriers at once, one needs to compensate for the phase change for all frequencies. Therefore, we rewrite transformation in the following way. Firstly, we remove the frequency shift

20 $$\begin{aligned} \tilde{q}_k^c(\omega ,z)= &\, {} \tilde{q}_k(\omega +2\lambda _k,z) \end{aligned}$$

21 $$\begin{aligned}= &\, \tilde{q}(\omega +2\lambda _k-2\lambda _k,z) e^{j t_k (\omega +2\lambda _k-\lambda _k)} \end{aligned}$$

22 $$\begin{aligned}= &\, \tilde{q}(\omega ,z)e^{j t_k (\omega +\lambda _k)}\text {.} \end{aligned}$$

Next, we replace $\lambda _k$ with $\lambda$ to compensate for the phase change for all frequencies.

23 $$\begin{aligned} \tilde{q}^c(\omega ,z)= &\, \tilde{q}(\omega ,z)e^{j 4 \lambda z (\omega +\lambda )} \end{aligned}$$

24 $$\begin{aligned}= &\, \tilde{q}(\omega ,z)e^{-j 2 \omega z (\omega -0.5\omega )} \end{aligned}$$

25 $$\begin{aligned}= &\, \tilde{q}(\omega ,z)e^{-j \omega ^2 z}\text {,} \end{aligned}$$

where $2\lambda =-\omega$ (the negative sign depends on the sign in the linear Fourier transform) and $\tilde{q}^c$ is $\tilde{q}$ after compensation. In principle we can apply the continuous frequency compensation to the serial scheme as well, however, the exponential calculation is computationally expensive and is not necessary for the serial case.

**Figure 7:**
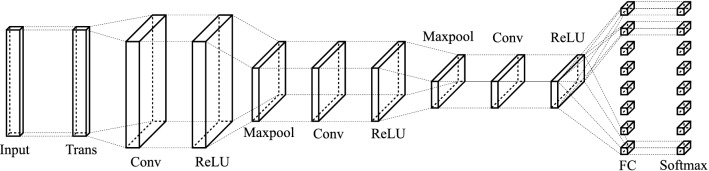
The conceptual schematic of the parallel network.

Now, the propagation phase is removed from the pulse, we come up with the following network scheme for multi-subcarrier simultaneous decoding. Figure 7 shows the design concept of the network. In comparison to the serial scheme, multiple fully connected layers are added in parallel to the last activation layer (ReLU) followed by softmax layers. A custom training process is used for this new scheme. The cross-entropy function is applied to all “softmax” outputs and the averaged cross-entropy is used as the loss value for calculating gradients for the next iteration. In the figure, only three convolutional layers are shown, but as the number of subcarriers increases, additional convolutional layers can be added if necessary as well as increase the number of filters in each layer.

**Figure 8:**
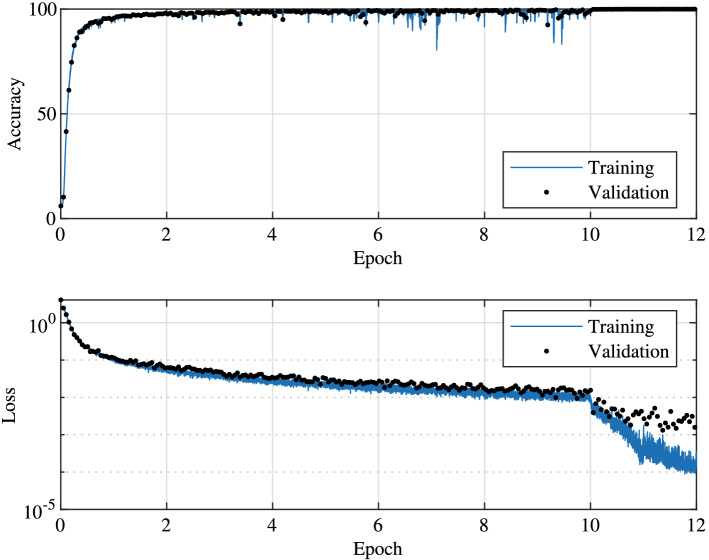
The accuracy and loss as functions of epochs for the parallel network scheme. An initial learning rate of 0.002 is used for epochs from 1 to 10 and increased to 0.0002 for epoch 11 and 0.00002 for epoch 12.

The training of the parallel network requires more train data than the serial scheme. For the example shown here, a total of 200,000 sample pulses were generated, 90% of which was used for training and 10% for validation. The convergence of the network is similar to the serial scheme. The training and validation accuracy and loss can be found in Fig. 8. The accuracy reaches 95% within the first epoch and reaches 99% before epoch 5. The initial learning rate for the first 10 epochs is 0.002. With this learning rate, we notice big accuracy fluctuations toward later epochs. The fluctuation is reduced immediately after reducing the learning rate to 0.0002 and the accuracy reaches 99.9% within the following epoch. A small gap between training and validation is observed. Further reducing the learning rate to 0.00002 results in a further reduction in training loss but the validation loss remains the same, a slight indication of over-fitting. We speculate a larger training data can help overcome the over-fitting and further improve the accuracy and loss of the network.

**Figure 9:**
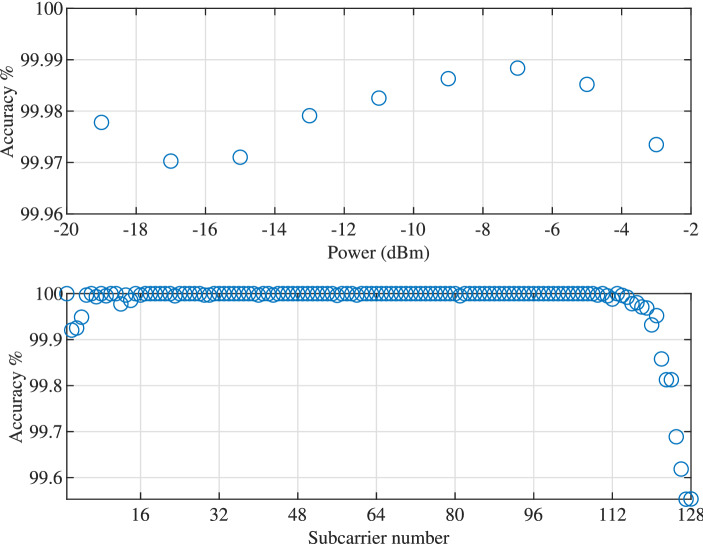
The accuracy for different pulse power and individual subcarrier.

The accuracy for different power ranges and individual subcarriers are shown in Fig. 9. The behaviours are rather similar to the serial network. The accuracy for different power ranges are rather close while the accuracy for the subcarriers on the edges of the spectrum is slightly lower than the majority.

The trained network of the parallel scheme occupies 128 MB of memory while the serial network occupies merely 0.5 MB. It is possible to improve the performance of the network by considering adding more hidden layers to the network or increasing the layer size. At the same time, it is also worth considering new network design concepts such as ResNet^50^, InceptionNet^51^ and SENet^52^ to get better overall performance with smaller network sizes. As the complexity of the network increases, one may also look into more advanced training methods, such as activated gradients method^53^, to improve the training performance for the network. In the networks presented here, all the convolutional layers contain 844*N* operations in total including 468*N* multiplications and 376*N* additions, where *N* is the input size. The operations in ReLU activation and max-pooling layers are negligible (about 2*N* operations). A fully connected layer has 32*N* operations with 16*N* multiplications and 16*N* additions. In the serial network, *N* = 64, but the computation is repeated *M* times in sequence with a total of $878\times N\times M$ operations. In the parallel network, *N* = 4096 and only one-pass computation is required. There are *M* fully connected layers in the multi-output stage resulting in a total of $(846+32\times M)\times N$ operations, which is about 2.8 times more operations than the serial scheme but the computation can be performed in parallel to save overall execution time.

## Conclusion

In this paper, we proposed two conceptual CNN schemes for decoding NFDM signals. A serial scheme that scarifies speed for small network size and a parallel scheme optimizes speed over size. The serial scheme only occupies 0.5 MB of memory space suitable for implementation on small computer chips for small users while the parallel scheme occupies 128 MB of memory which requires more expansive hardware for usages in places such as data centres. Both network schemes have been demonstrated by training with simulated data and been able to reach more than 99.9% accuracy.

This work only demonstrates conceptual designs based on simulation of pulse propagation in optical fibres. To implement the proposed CNN scheme for practical experimentation, additional adjustments and modifications may be required. In practice, for instance, an unknown initial phase may present in the signal, which can slowly drift over time. This initial phase can be seen as a rotation of the constellation of symbol $c_n$. In this work, the simulated training data have zero initial phases. However, this phase information is contained inside each signal burst and thus can potentially be identified using a CNN as well. For future work, network design shall take the initial phase into account either through a separate network or adding layers to the current designs to make the network more suitable for practical use. Demonstrations of the proposed schemes using experimentally collected data will also be considered in the next stage of development.

## Methods

The CNN models in this work are developed using Matlab’s Deep Learning Toolbox (version R2020a), but the same results can be reproduced using other deep learning frameworks such as TensorFlow and PyTorch. In each network, the input layer is a *N*-row long by 2-column wide array, where each column corresponds to the real and imaginary parts of the signal. Number *N* is the number of sampling points across a subcarrier segment of pulse spectrum in the serial scheme or the full spectrum of the pulse in the parallel scheme. The first convolution layer has a kernel size of 3 $\times$ 3, a stride step size of 1, a padding size of 1 and no dilation. The rest of the convolution layers are the same except a kernel size is reduced to 3 $\times$ 1. The first max-pooling layer has a pool size of 2 $\times$ 2, a stride step size of 2 and no padding. For the rest of the max-pooling layers, the pool size is reduced to 2 $\times$ 1. Each fully connected layer has an output size of 16 corresponding to the 16-QAM used in this work, which is mapped to values from 0 to 15 directly and used as labels for each input. Adam optimizer was used for training the network. The decay rate of gradient and squared gradient moving averages are set to be 0.9 and 0.99, respectively. L2 regularization is applied with a factor of 0.00002. The initial learning rate is set to 0.002 and then reduced manually each time by a factor of 10 during the training process. More details can be found in corresponding sections where the network training results are discussed.

The key information of training data generation is given in the data generation section, in which, the pulse propagation over a standard single-mode optical fibre is simulated by solving the following general nonlinear Schrödinger equation using a split-step Fourier method^54^,

26 $$\begin{aligned} \frac{\partial }{\partial z}q(z,t) + \frac{\alpha }{2}q(z,t) - \sum _{k\ge 2}\frac{i^{k+1}}{k!}\beta _k\frac{\partial ^k}{\partial t^k}q(z,t) = i\gamma \left( 1 + i\tau _0\frac{\partial }{\partial t}\right) \left( q(z,t)\int _{-\infty }^{\infty }R(t^\prime ) \times \left| q(z,t-t^\prime )\right| ^2 dt^\prime )\right) \text {,} \end{aligned}$$

in which $R(t)=(1-f_R)\delta (t)+f_R h_R(t)$, $\displaystyle h_R(t)=\frac{\tau _1^2+\tau _2^2}{\tau _1\tau _2^2}\exp (-\frac{t}{\tau _2}) \sin (\frac{t}{\tau _1})$, $f_R=0.18$, $\tau _0=0.82$ fs, $\tau _1=12.2$ fs, $\tau _2=32$ fs. The fibre loss coefficient $\alpha =0.0461$ km$^{-1}$, dispersion $\beta _2=-21.7$ ps$^2$/km, $\beta _3=18.6$ ps$^3$/km and nonlinearity $\gamma =1.1$ W$^{-1}$ km$^{-1}$ . In the split-step method, 2048 sampling points are used in the time window with a sampling rate of 96 GS/s. The propagation step size is set to 10 m per step. After each span of propagation, the pulses are amplified to compensate the loss during the propagation through a scaling factor $\exp (\frac{\alpha }{2}L)$, where $L=50$ km is the span length. A white Gaussian noise of − 40 dBm is added every time the signal is amplified.

## Supplementary Information

Supplementary Information.
