# Supplementary material for: Serial and parallel convolutional neural network schemes for NFDM signals
Source: Sci Rep. 2022 May 13;12:7962. doi: 10.1038/s41598-022-12141-4 (PMC9106738; doi:10.1038/s41598-022-12141-4)
Supplement: Supplementary file 1 — Supplementary Information. [file 41598_2022_12141_MOESM1_ESM.pdf]

Serial and parallel convolutional neural network schemes for NFDM signals: supplementary material

Wen Qi Zhang[1,*], **Terence H. Chan**[2], **and Shahraam Afshar V.**[1]

[1]Laser Physics and Photonic Devices Laboratories, School of Engineering, University of South Australia, Australia
[2]Institute for Telecommunications Research, University of South Australia, Australia
[*]wenqi.zhang@unisa.edu.au

ABSTRACT

This document provides supplementary material to support the journal submission titled "Serial and parallel convolutional neural network schemes for NFDM signals".

Feature visualization

The features learned by the convolutional layers of the serial network (shown in main document Fig. 4) are visualized in Fig. 1 using deep dreaming[1]. In each of Fig. 1(a∼d), the x-axis and y-axis correspond to linear frequency and signal amplitude (both real and imaginary parts), respectively. The first convolutional layer, shown in Fig. 1(a), seems to detect low frequency components of the input signal. As the layers get deeper, the convolutional layers detect more complex patterns with higher frequencies in the input signal.

References

1. Olah, C., Mordvintsev, A. & Schubert, L. Feature visualization. *Distill*. DOI: 10.23915/distill.00007 (2017). Https://distill.pub/2017/feature-visualization.

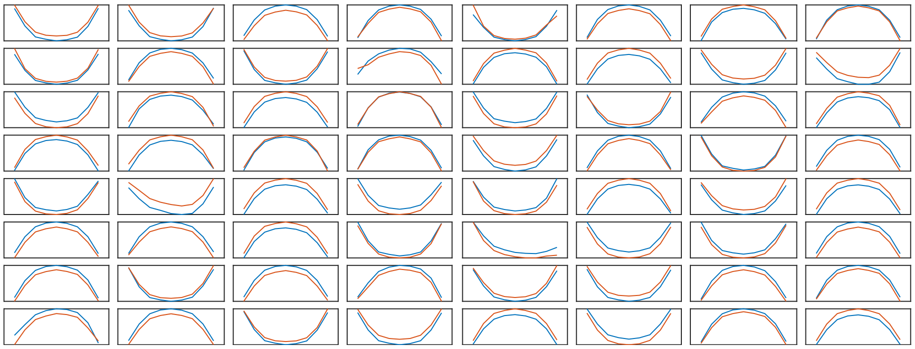

(a) First convolutional layer

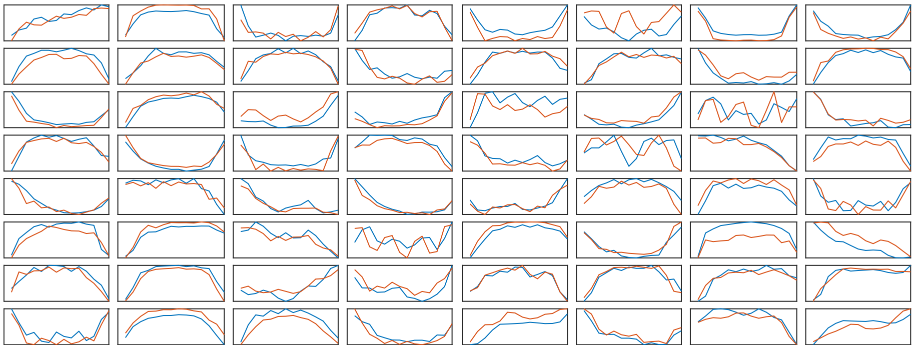

(b) Second convolutional layer

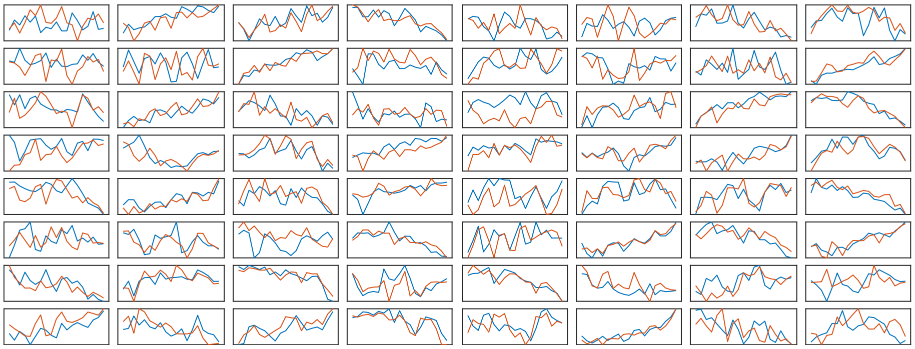

(c) Third convolutional layer

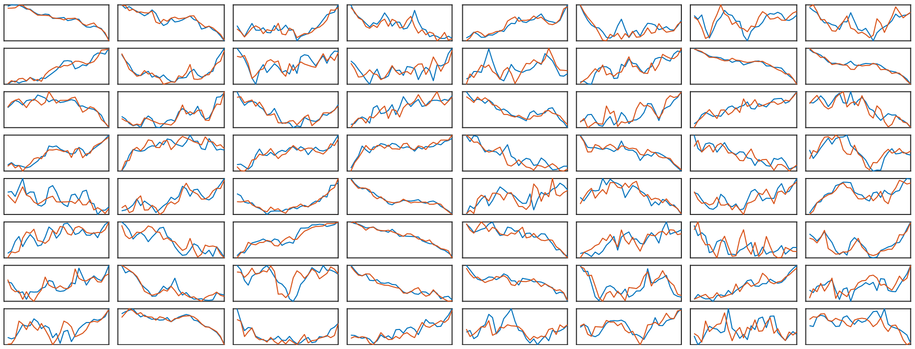

(d) Fourth convolutional layer

Figure 1. The learnt features of the four convolutional layers of the serial network visualized using deep dream. The x and y axes correspond to linear frequency and signal amplitude of both real (red curves) and imaginary (blue curves) parts, respectively.
